# A predictive model of the temperature-dependent inactivation of coronaviruses

**DOI:** 10.1063/5.0020782

**Published:** 2020-08-11

**Authors:** Te Faye Yap, Zhen Liu, Rachel A. Shveda, Daniel J. Preston

**Affiliations:** Department of Mechanical Engineering, Rice University, 6100 Main Street, Houston, Texas 77005, USA

## Abstract

The COVID-19 pandemic has stressed healthcare systems and supply lines, forcing medical doctors to risk infection by decontaminating and reusing single-use personal protective equipment. The uncertain future of the pandemic is compounded by limited data on the ability of the responsible virus, SARS-CoV-2, to survive across various climates, preventing epidemiologists from accurately modeling its spread. However, a detailed thermodynamic analysis of experimental data on the inactivation of SARS-CoV-2 and related coronaviruses can enable a fundamental understanding of their thermal degradation that will help model the COVID-19 pandemic and mitigate future outbreaks. This work introduces a thermodynamic model that synthesizes existing data into an analytical framework built on first principles, including the rate law for a first-order reaction and the Arrhenius equation, to accurately predict the temperature-dependent inactivation of coronaviruses. The model provides much-needed thermal decontamination guidelines for personal protective equipment, including masks. For example, at 70 °C, a 3-log (99.9%) reduction in virus concentration can be achieved, on average, in 3 min (under the same conditions, a more conservative decontamination time of 39 min represents the upper limit of a 95% interval) and can be performed in most home ovens without reducing the efficacy of typical N95 masks as shown in recent experimental reports. This model will also allow for epidemiologists to incorporate the lifetime of SARS-CoV-2 as a continuous function of environmental temperature into models forecasting the spread of the pandemic across different climates and seasons.

The COVID-19 pandemic has overwhelmed medical facilities worldwide and caused a shortage of typically disposable personal protective equipment (PPE), forcing medical workers to reuse or work without proper PPE.^1,2^ Researchers have explored decontamination procedures that might allow PPE to be reused safely,^3,4^ and medical workers have begun implementing these procedures, including decontaminating disposable masks with ultraviolet (UV) irradiation.^5^ However, UV decontamination faces several drawbacks, including an inability to kill viruses trapped within crevices that are not illuminated and a lack of availability at clinics in low-income areas and in most peoples' homes.^6^ Alternative methods of decontamination, namely, steam sterilization, alcohol washing, and bleach washing, are useful for glassware and other durable materials, but have been reported to degrade single-use PPE.^4,7,8^ On the other hand, dry heat decontamination can be performed almost anywhere (including home ovens and rice cookers) and inactivates viruses within crevices without damaging the delicate PPE.^7,9,10^ However, dry heat decontamination guidelines for SARS-CoV-2 remain limited to a few experimental measurements constrained to specific temperatures that do not apply to all heating devices.^11^

Meanwhile, virus transmission has been linked to variations in outdoor climate, where colder atmospheric temperatures lead to longer virus lifetimes outside of hosts. This effect has been reported for influenza,^12,13^ the common cold,^14^ SARS-CoV-2,^11,15^ SARS-CoV-1,^16,17^ and MERS-CoV.^18,19^ Even at a local scale, a recent resurgence of COVID-19 cases in a seafood market was linked to low temperatures.^20^ Epidemiologists would benefit from knowledge of the lifespan of SARS-CoV-2 as a continuous function of the atmospheric temperature to accurately model the spread of COVID-19. Furthermore, understanding the temperature-dictated inactivation time could help predict the resurgence of cases as colder weather returns to the Northern Hemisphere, following a similar trend to that of the seasonal flu.^21^

We introduce an analytical model based on the rate law for a first-order reaction and the Arrhenius equation that enables prediction of the thermal inactivation rate and lifetime of coronaviruses, including SARS-CoV-2, as a function of temperature. These viruses are treated as macromolecules undergoing thermal denaturation; we confirm that coronaviruses undergo thermal denaturation because their inactivation behavior follows the Meyer–Neldel rule.^22^ The time required to achieve the desired log-scale reduction in viable virions (e.g., by a factor of 10^3^ as typically used for viral decontamination^23–26^) was used to generate dry heat decontamination guidelines for SARS-CoV-2 relevant to temperature ranges accessible in commonly available heating devices. The model also predicts the lifetime of human coronaviruses as a continuous function of temperature in various climates, which will assist epidemiologists in understanding the regionally dependent lifetime of the SARS-CoV-2 virus, as well as the potential of a COVID-19 resurgence in autumn and winter.

Reports in the literature provide abundant data to construct a predictive analytical model capturing the thermal effects on virus inactivation. We specifically focused on the inactivation of coronaviruses, a group of enveloped viruses often responsible for respiratory or gastrointestinal diseases in mammals and birds.^27^ We compiled hundreds of data points for the inactivation of five coronaviruses, with subdivisions based on (i) strains of each virus, (ii) environmental pH levels, and (iii) relative humidity (RH) conditions, resulting in 14 datasets [Fig. 1(a)]. These viruses include (i) Severe Acute Respiratory Syndrome Coronavirus (SARS-CoV-1 and SARS-CoV-2);^11,17,28–30^ (ii) Middle East Respiratory Syndrome Coronavirus (MERS-CoV);^18,19^ (iii) Transmissible Gastroenteritis Virus (TGEV);^31^ (iv) Mouse Hepatitis Virus (MHV);^32,33^ and (v) Porcine Epidemic Diarrhea Virus (PEDV).^34^

**FIG. 1.**
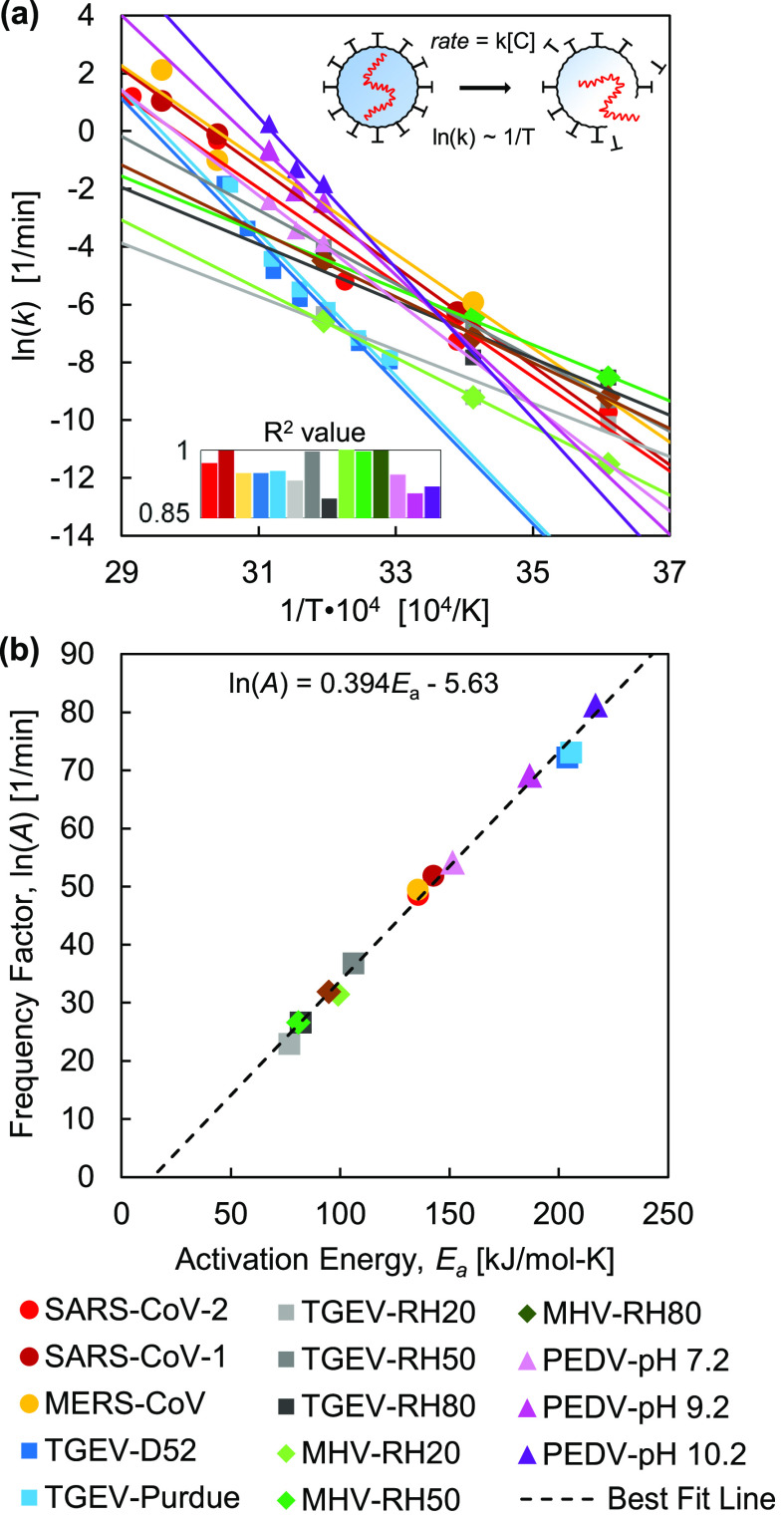
Thermal inactivation behavior of coronaviruses. An Arrhenius plot (a) shows the dependence of inactivation rate constant on temperature for the coronaviruses. Each coronavirus dataset was fitted using linear regression [Eq. (2)], where the inserted chart presents the R^2^ values for the linear fits. The resulting activation energy, E_a_, and frequency factor, ln(A), were back-calculated from each linear fit according to Eq. (2) and plotted (b); the linear correlation between ln(A) and E_a_ indicates protein denaturation.^22,41^

The rate law describes the inactivation behavior of microbes.^35^ Non-first-order rate laws have been applied to the inactivation of some microbes,^36–38^ particularly bacteria with heterogeneous populations,^39^ but the inactivation of most viruses—including the coronaviruses considered in our analysis—follows a first-order reaction, with viable virions as reactants and inactivated virions as products [Eq. (1)]:

$$\left[C\right]=[C_{0}]e^{-kt}.$$ (1)

The majority of primary experimental data for the inactivation of viruses is reported in plots of the log of concentration, ln([C]), as a function of time, *t*, with [C_0_] being the initial concentration of viable virions. We applied a linear regression to each set of primary data to determine the rate constant, *k*, for the inactivation of a virus at a given temperature, *T*, determined by calculating the slope, *k* = −Δln([C])/Δ*t*. Each of these pairs of (*k*, *T*) yields one data point in Fig. 1(a), with details in the supplementary material, Figs. S1–S28.

Virus inactivation occurs due to thermal denaturation of the proteins that comprise each virion. The temperature dependence of the thermal denaturation process is captured by the Arrhenius equation,^40^ which yields a linear relationship between ln(*k*) and 1/*T* [Eq. (2)]:

$$\ln\left(k\right)=-\frac{E_{a}}{RT}+\ln\left(A\right),$$ (2)

where *R* is the gas constant, *E_a_* is the activation energy associated with the inactivation of the virus (i.e., the energy barrier to be overcome for protein denaturation), and *A* is the frequency factor. In Fig. 1(a), ln(*k*) and 1/*T* are plotted according to the Arrhenius equation [Eq. (2)]. The activation energy, *E_a_*, and the natural log of the frequency factor, ln(*A*), can be obtained by equating –*E*_a_/*R* and ln(*A*) from Eq. (2) with the slopes and intercepts from the linear fits in Fig. 1(a), respectively, and are plotted in Fig. 1(b). The linear correlation between ln(*A*) and *E_a_* indicates that coronaviruses undergo a thermal denaturation process following the Meyer–Neldel rule,^22^ supporting our hypothesis that they are primarily inactivated by the thermally driven degradation of proteins. In fact, the linear regression calculated in this work, [ln(*A*) = 0.394*E*_a_ − 5.63], is nearly identical to those calculated in two prior studies on the denaturation of tissues and cells, which report [ln(*A*) = 0.380*E*_a_ − 5.27]^22^ and [ln(*A*) = 0.383*E*_a_ − 5.95].^41^

The degree of inactivation of a pathogen is defined by the ratio of the concentration (amount) of a pathogen to its initial concentration, [C]/[C_0_], often in terms of orders of magnitude; an *n*-log inactivation refers to a reduction in the concentration of 10 raised to the *n*th power ([C]/[C_0_] = 10^−*n*^). Equations (1) and (2) combine to yield an analytical model used in determining the time required to achieve an *n*-log reduction in a pathogen [Eq. (3)]:

$$t_{n-\log}=-\frac{1}{A}e^{\left(\frac{E_{a}}{RT}\right)}\ln\left(10^{-n}\right).$$ (3)

The U.S. Food and Drug Administration recommends a 3-log (99.9%) reduction in the number of virions for the decontamination of non-enveloped viruses (i.e., [C]/[C_0_] = 10^−3^).^23–26,42,43^ Since non-enveloped viruses are shown to be more resilient to elevated environmental temperatures than their enveloped counterparts (including coronaviruses),^44,45^ we refer to the time required to achieve a 3-log reduction as the coronavirus *lifetime*, indicative of a conservative prediction for both decontamination time and viable lifetime outside a host. The time required to achieve an *n*-log reduction is directly proportional to the *n* value; therefore, a more conservative decontamination time could be obtained by inserting a different value of *n* into Eq. (3), which would change the *n-*log reduction predictions of lifetime by a multiplicative factor of *n_desired_*/*n_current_* (e.g., in this work, *n_current_* = 3; therefore, a 6-log reduction would require doubling of the lifetimes predicted in this work).

Figure 2 reports the virus lifetimes generated from Eq. (3) as a function of temperatures ranging from room temperature to temperatures achievable using common heating devices. In Fig. 2(a), all five types of coronaviruses are plotted to show the variation across different environmental temperatures. The plot in Fig. 2(b) shows similar data, with the exception of data from Casanova, *et al.*,^16^ due to the possible experimental error in the primary data (see the supplementary material, Sec. S3), and with the lifetime axis scaled linearly to highlight the exponential dependence of lifetime on temperature. The human coronaviruses SARS-CoV-2 and SARS-CoV-1 exhibit a similar trend in thermal degradation, in agreement with recent work.^30^ We observed that SARS-CoV-2 has a slightly longer mean lifetime than SARS-CoV-1 outside a host, potentially contributing to its relatively high reproduction number, *R*_0_. However, based on uncertainty analysis, Fig. 3 indicates that the prediction intervals (PIs) of SARS-CoV-1 and SARS-CoV-2 overlap, suggesting that additional data would be needed to definitively support the conclusion that SARS-CoV-2 has a longer lifetime. The prediction interval is used to estimate the variation in coronavirus lifetimes predicted by the analytical model. The prediction interval can account for uncertainties corresponding to different virus strains due to genetic mutations, as well as variations in experimental conditions, such as RH and fomites, and a conservative estimate of the maximum lifetime of a coronavirus given this uncertainty can be determined with different levels of confidence (90%, 95%, and 97.5% prediction intervals are shown in Fig. 3). The details of statistical uncertainty for all of the viruses are included in the supplementary material, Table S3. The average lifetime for the human coronaviruses SARS-CoV-2 and SARS-CoV-1 is shown in Table I. The temperature values displayed in the table illustrate both (i) common environmental temperatures and (ii) temperatures appropriate for thermal decontamination.

**FIG. 2.**
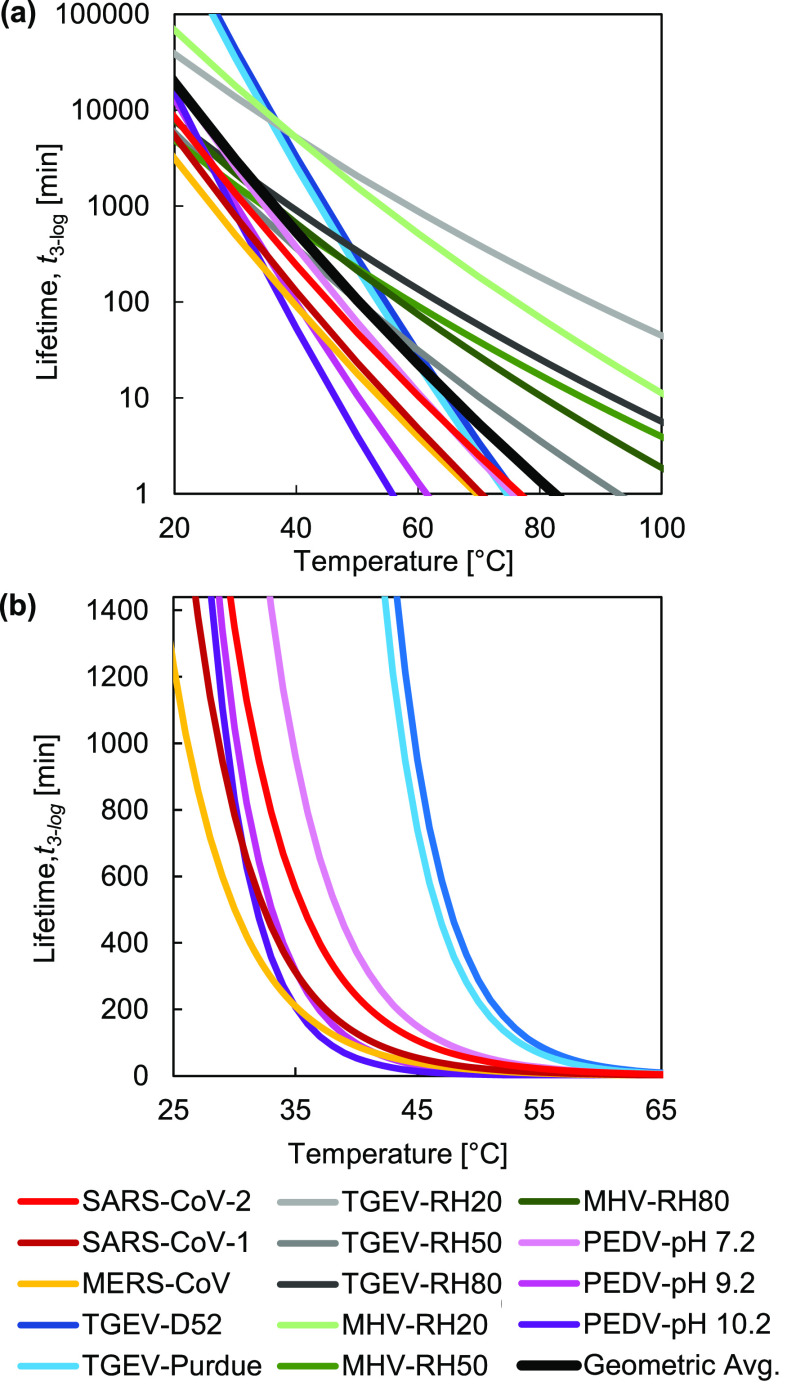
Virus lifetime as a function of temperature. Predictions are shown in (a) for all the coronaviruses analyzed in this work, with the average lifetime presented in black. All coronaviruses, excluding the data sourced from Casanova, *et al.*, are replotted in (b) with a linearly scaled vertical axis (1440 min = 1 day) to highlight the exponential dependence of lifetime on temperature.

**FIG. 3.**
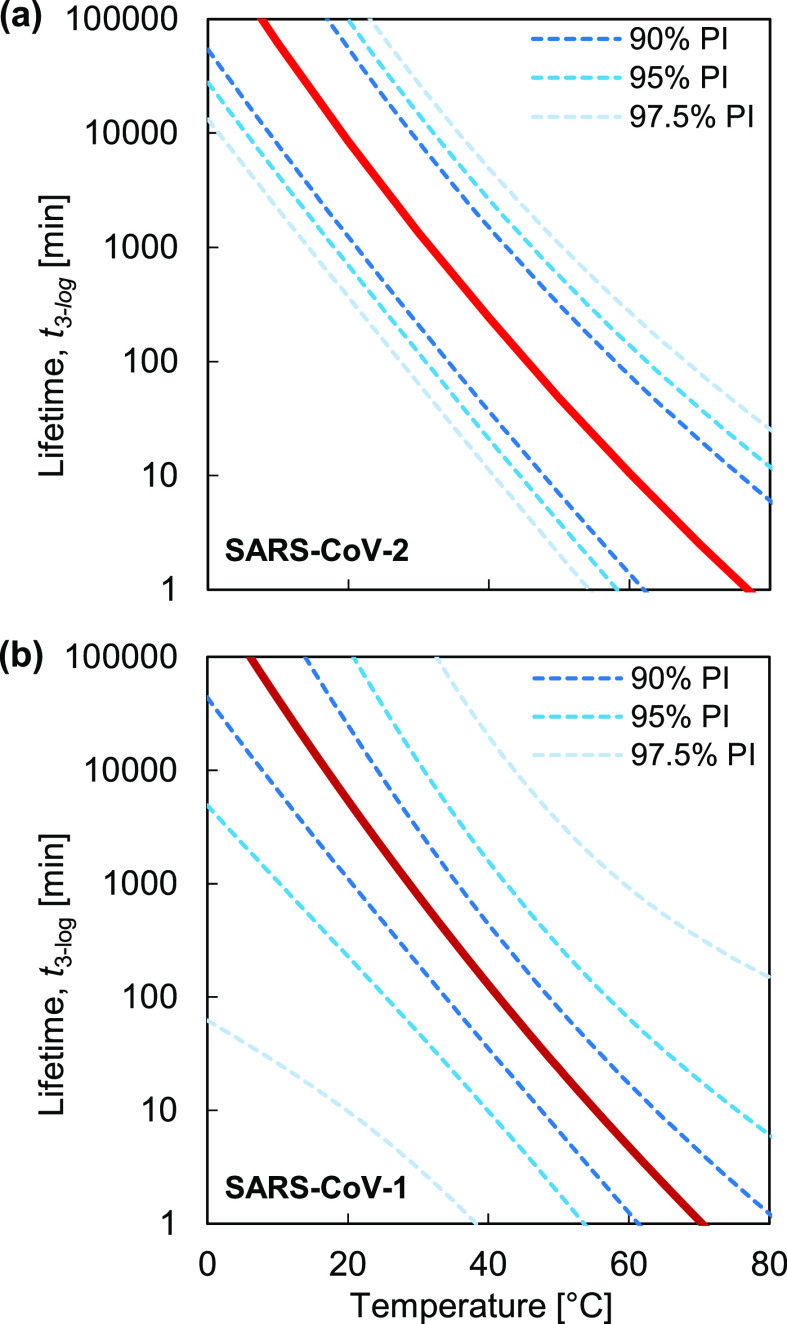
The lifetimes of SARS-CoV-2 (a) and SARS-CoV-1 (b) are highlighted, and 90%, 95%, and 97.5% prediction intervals (PIs) are used to illustrate uncertainties in the predicted lifetimes based on statistical analysis.

**TABLE I.** The average lifetimes for SARS-CoV-2 and SARS-CoV-1 across a range of environmental and decontamination temperatures. The upper limit of a 95% prediction interval based on statistical analysis of the data is included in parentheses as a conservative estimate of the maximum lifetime across different mutations and environmental conditions. The mean lifetimes of all human coronaviruses considered in this work were greater than one month at temperatures below 10 °C.

	Temperature	SARS-CoV-2 lifetime, *t*_3-log_	SARS-CoV-1 lifetime, *t*_3-log_
Environmental temperatures	10 °C	>1 month	29.8 d *(> 1 month)*
	15 °C	15.5 d *(>1 month)*	10.4 d *(> 1 month)*
	20 °C	5.9 d *(>1 month)*	3.8 d *(> 1 month)*
	25 °C	2.3 d *(25.5 d)*	1.4 d *(25.4 d)*
	30 °C	22.5 h *(10.0 d)*	13.1 h *(8.26 d)*
	35 °C	9.4 h *(4.2 d)*	5.2 h *(2.9 d)*
	40 °C	4.0 h *(1.8 d)*	2.1 h *(1.1 d)*
Decontamination	60 °C	10.5 min *(2.3 h)*	4.8 min *(1.1 h)*
	70 °C	2.5 min *(38.6 min)*	1.1 min *(18.4 min)*
	80 °C	<1 min *(11.9 min)*	<1 min *(6.1 min)*
	90 °C	<1 min *(4.0 min)*	<1 min *(2.3 min)*

We estimated the regional lifetime of SARS-CoV-2 based on climate temperatures in the United States. We used temperatures averaged over January to March, 2020, corresponding to the onset of the COVID-19 pandemic [Fig. 4(a)], and July to September, 2019, as a rough prediction of SARS-CoV-2 lifetimes in summer 2020 [Fig. 4(b)]. Summer weather in the Northern Hemisphere will reduce SARS-CoV-2 outdoor-lifetime significantly, potentially slowing the transmission of COVID-19. The predictions in Fig. 3 are based on a constant temperature profile and do not account for daily temperature fluctuations, which may result in shorter lifetimes than predicted due to the exponential dependence of the reaction rate on temperature. Additional environmental effects, like UV from sunlight, may further reduce inactivation time; with these limitations in mind, Fig. 4 represents a conservative prediction of SARS-CoV-2 lifetime across the United States, and lifetimes greater than one month are not reported.

**FIG. 4.**
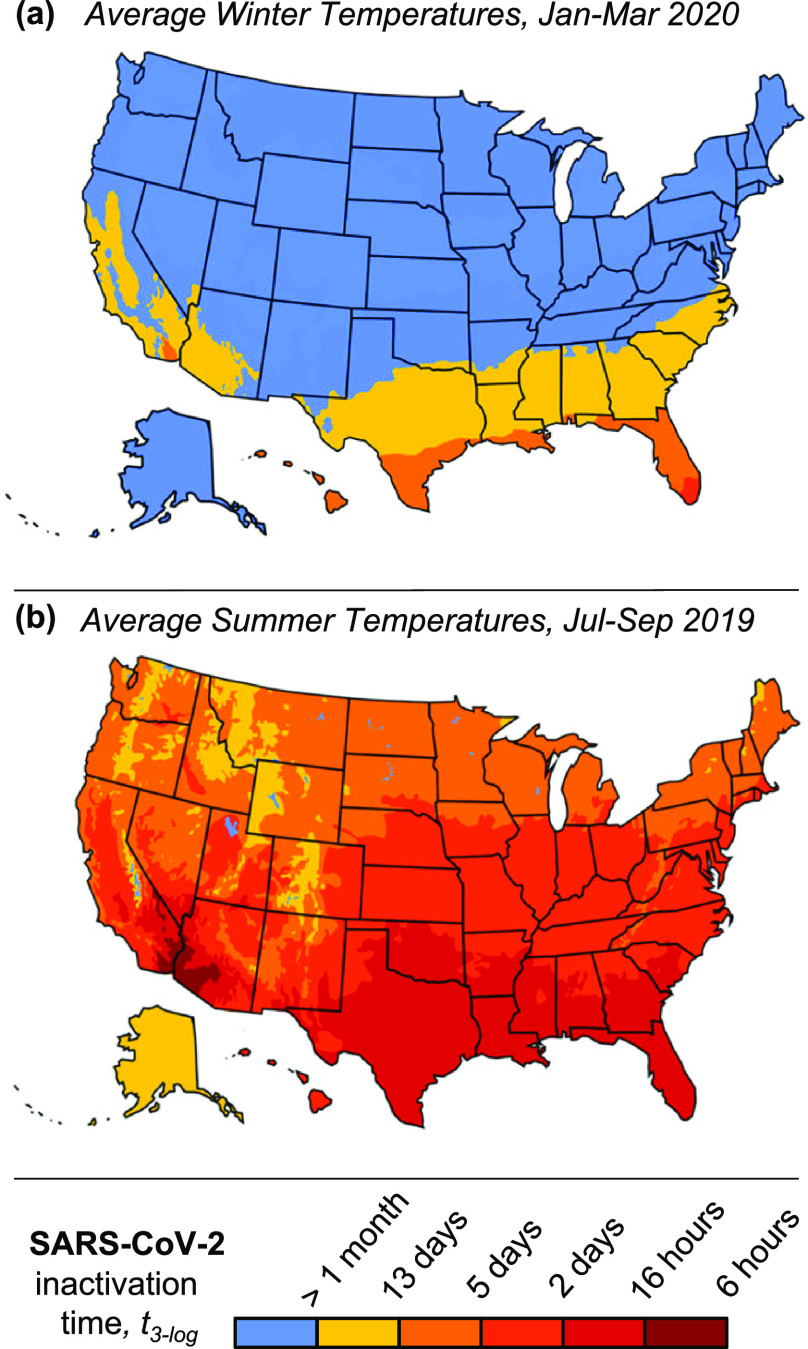
Lifetime of SARS-CoV-2 outside of a host across the United States in winter and summer. Predictions are based on (a) average temperature data from January to March, 2020 (corresponding to the onset of the COVID-19 pandemic) and (b) average temperature data from July to September, 2019 (to show characteristic lifetimes in summer). Temperatures are reported in Figures S34 and S35.

We tested the predictive ability of the thermodynamic model presented here by comparing the results to experimental data that had not been used to train the model. SARS-CoV-1 was reported to require 5 days at room temperature to achieve a 5-log reduction;^46^ our model predicts an inactivation time of 4.2 days under the same conditions. In another report, SARS-CoV-1 was heated to 56 °C and required only 6 min to achieve a 6-log reduction;^29^ our model predicts a time of 17 min. A third report claimed that SARS-CoV-1 required 30 min to achieve an approximately 6-log reduction at 60 °C;^47^ our model predicts a time of 10 min. A more recent report shows that both SARS-CoV-2 and SARS-CoV-1 require 72 h for a 3-log reduction on plastic surfaces maintained around 23 °C; our model predicts lifetimes of 80 and 50 h, respectively, in good agreement with the reported data.^30^ All of these reported lifetimes were within the uncertainty bounds of the model predictions. Considering the similarity in inactivation behavior for SARS-CoV-1 and SARS-CoV-2,^30^ validation with SARS-CoV-1 suggests that this model will be a useful tool to estimate the lifetime of SARS-CoV-2.

The model is limited to temperature-based predictive ability and does not consider relative humidity or the fomite (i.e., the surface material on which a virion rests), both of which appear to affect inactivation times.^11,16,30,48^ Variations in lifetime at a given temperature due to these environmental factors can be interpreted as catalytic effects;^49^ incorporating a corresponding adjustment to the activation energy might enable additional predictive capabilities. Another limitation of this model is its reliance on a limited set of primary data, which may contain experimental error (all primary data are reproduced in the supplementary material); statistical prediction uncertainties are described in the supplementary material, Sec. S5. In addition, this model assumes that the enthalpy and entropy of the inactivation reaction are constant as temperature changes. This assumption is typically valid for macromolecules such as proteins.^22^ Some reports suggest that multiple inactivation reaction pathways can occur near room temperature, but these reports are limited in scope and do not agree with each other, and further work would need to be done before considering or implementing such effects.^31,40^ Finally, the extrapolation of our model to higher temperatures outside the range of the primary data (e.g., above 100 °C) may be unfounded if alternate inactivation reaction pathways become available at these elevated temperatures.

Fortunately, the results in Table I indicate that dry heat decontamination is feasible for inactivation of all types of coronaviruses, including SARS-CoV-2. The most common material used in surgical masks and N95 respirators is non-woven polypropylene,^50,51^ which can be decontaminated with dry heat below its melting point (156–168 °C).^52,53^ Cui and colleagues show that thermal cycling (75 °C, 30 min heating, applied over 20 cycles) does not degrade the filtration efficiency of N95-level facemasks,^9^ and Lin *et al.* report no significant degradation in the effectiveness of surgical masks after heating to 160 °C for 3 min.^7^ Therefore, we expect that dry heat decontamination is an effective decontamination method, while also feasible within relatively short times (conservatively, less than 40 min at 70 °C; Table I) and achievable by the majority of people with access to home ovens, rice cookers, or similar inexpensive heating devices.

In summary, this work provides guidelines to medical professionals and the general public for the effective, safe thermal decontamination of PPE. In addition, the sensitivity of coronaviruses to environmental temperature variations, shown in Table I and Fig. 4, indicates that the thermal inactivation of SARS-CoV-2 must be considered in epidemiological studies predicting its global spread and, potentially, seasonal recurrence; our model can be incorporated in these studies due to its ability to predict virus lifetime as a continuous function of environmental temperature. Finally, the modeling framework presented here offers a fundamental understanding of virus thermal inactivation that can help fight the COVID-19 pandemic, as well as future outbreaks of other coronaviruses.

See the supplementary material for primary datasets for each virus studied in this work,^54–56^ tables of activation energy and frequency factor calculated from the data, temperature data in the United States corresponding to winter and summer, and details on the statistical analysis and uncertainty in predictions.^57^

## AUTHORS' CONTRIBUTIONS

T.F.Y. and D.J.P. compiled and analyzed the data and developed the analytical model. All authors contributed to the interpretation of results and writing and editing the manuscript. D.J.P. guided the work. Z.L. and R.A.S. contributed equally to this work.

## Data Availability

The data that support the findings of this study are available within the article and its supplementary material.
